# Reliability of the E-test and VITEK 2 Compact for Tigecycline Susceptibility Testing in Clinical Acinetobacter baumannii Isolates Compared With Broth Microdilution

**DOI:** 10.7759/cureus.104860

**Published:** 2026-03-08

**Authors:** Amresh Pati, Bilal Ahmad Malik, Kavita Gupta, Ashoka Mahapatra

**Affiliations:** 1 Microbiology, Kalinga Institute of Medical Sciences, Bhubaneswar, Bhubaneswar, IND; 2 Microbiology, Government Medical College, Kathua, Kathua, IND; 3 Microbiology, Post Graduate Institute of Child Health, Noida, Noida, IND; 4 Microbiology, All India Institute of Medical Sciences, Bhubaneswar, Bhubaneswar, IND

**Keywords:** acinetobacter baumannii, broth microdilution, e test, tigecycline, vitek 2 compact

## Abstract

Background: The growing incidence of multidrug-resistant *Acinetobacter baumannii *(MDR AB) has left very limited therapeutic options. Tigecycline, the first antibiotic in the glycylcycline class, is approved by the U.S. Food and Drug Administration (US FDA) for the treatment of complicated intra-abdominal infections and complicated skin and skin structure infections. This is one of the few last-resort options for infections caused by MDR AB. However, there is a lack of officially available breakpoints for *Acinetobacter baumannii *against tigecycline.

Objective: This study aims to determine the tigecycline susceptibility of clinical *A. baumannii* isolates by broth microdilution (BMD), E-test, and VITEK 2 Compact, and to evaluate the performance of E-test and VITEK 2 Compact against BMD as the reference standard.

Methodologies: A total of 150 clinical isolates of *A. baumannii *from sputum, blood, urine, pus, and sterile body fluids identified by VITEK 2 Compact from May 2019 to June 2021 were included in this study. Tigecycline susceptibility was determined by BMD, E-test (minimum inhibitory concentration (MIC): 0.016-256 µg/mL), and VITEK 2 Compact, with MICs interpreted using EUCAST (European Committee on Antimicrobial Susceptibility Testing) 2020 *Enterobacterales *breakpoints.

Result: The proportion of isolates categorized as susceptible by BMD was 90.67%, with MIC_50_ as 0.125 µg/mL, whereas MIC_50_ was higher for E-test and VITEK 2 Compact, having values of 0.75 µg/mL and 1 µg/mL, respectively. MIC_90_ was found to be 0.5 µg/mL in BMD, whereas higher values of 1 µg/mL and 4 µg/mL were found in E-test and VITEK 2 Compact, respectively.

Conclusion: Discordant results have been observed across different susceptibility testing methods as well as using different interpretative criteria. As this study was conducted on a small number of isolates, additional studies are needed to determine an interpretation category for Indian isolates.

## Introduction

*Acinetobacter baumannii *(*A. baumannii*) is a well-recognized emerging pathogen in intensive care units (ICUs) [1]. Its ability to survive for prolonged periods in hospital settings and its rapid acquisition of antimicrobial resistance have led to its emergence as a major multidrug-resistant (MDR) pathogen in healthcare settings. Carbapenems were once considered the drugs of choice for the treatment of MDR *A. baumannii*; however, their effectiveness has declined due to the increasing prevalence of carbapenem resistance [2].

Currently, only limited therapeutic options remain for the treatment of MDR *A. baumanii* (MDRAB) infections, primarily tigecycline and polymyxins. However, several studies have reported that colistin monotherapy may be associated with nephrotoxicity and may also contribute to the emergence of resistance [2,3].

Tigecycline, a synthetic glycylcycline antibiotic, demonstrates potent in vitro activity against a wide range of MDR bacterial pathogens, including MDRAB, and is considered a last-line therapeutic option for severe infections.

The U.S. Food and Drug Administration (US FDA) has approved tigecycline for the treatment of complicated intra-abdominal infections and complicated skin and soft tissue infections [4]. Its mechanism of action involves inhibition of bacterial protein synthesis through binding to the 30S ribosomal subunit during translation, and it remains active against many tetracycline resistance mechanisms [5].

Despite its therapeutic importance, non-susceptibility to tigecycline is increasingly being reported, partly due to its extensive use without adequate antimicrobial susceptibility testing (AST). Susceptibility testing for tigecycline is challenging because interpretative breakpoints for *A. baumannii* are not defined in current CLSI (Clinical and Laboratory Standards Institute), EUCAST (European Committee on Antimicrobial Susceptibility Testing), or FDA guidelines [6]. Furthermore, antimicrobial susceptibility results for tigecycline may vary depending on the testing methodology. Disk diffusion and E-test methods are considered less reliable for tigecycline susceptibility testing, with several studies reporting lower susceptibility rates than those observed with the reference broth microdilution (BMD) method [7,8].

Therefore, the present study aimed to determine tigecycline susceptibility among clinical isolates of *A. baumannii* using available phenotypic methods (BMD, E-test, and VITEK 2 Compact) and to evaluate the performance of E-test and VITEK 2 Compact against BMD as the reference standard.

## Materials and methods

A total of 150 non-duplicate clinical isolates of *A. baumannii *obtained from various clinical specimens, such as respiratory samples (81), blood (25), urine (12), pus and wound exudates (8), and other sterile body fluids (24), submitted for routine diagnostic testing between May 2019 and June 2021, were included in the study. The isolates were initially identified using the VITEK 2 Compact and subsequently confirmed by detection of the intrinsic *blaOXA-51* gene using conventional polymerase chain reaction (PCR). *A. baumannii* ATCC 19606 was used as the reference strain. The susceptibility of *A. baumannii* isolates to tigecycline was determined using three methods: BMD, E-test, and VITEK 2 Compact.

BMD was performed in a 96-well U-bottom microtiter plate containing a final volume of 100 µL comprising 50 µL of double-strength cation-adjusted Mueller-Hinton broth (CA-MHB), 25 µL of tigecycline solution, and 25 µL of bacterial inoculum. Tigecycline powder (Sigma, St. Louis and Burlington, MA; European Pharmacopeia Reference Standard, Code: Y0001961, Batch: 1.0) was dissolved in DMSO to prepare a 1 mg/mL stock solution, and appropriate 4× working concentrations were prepared freshly on the day of testing due to oxygen sensitivity. Twofold serial dilutions were made to obtain final concentrations ranging from 4 to 0.12 µg/mL. The bacterial suspension was prepared from 18-24 h colonies, adjusted to 0.5 McFarland, and further diluted to achieve a final inoculum of 5 × 10^8^ CFU/mL in each well.

As CLSI has not established interpretative MIC (minimum inhibitory concentration) breakpoints for tigecycline against *A. baumannii*, the EUCAST 2020 breakpoints recommended for *Enterobacterales* (susceptible (S) ≤0.5 µg/mL; resistant (R) >0.5 µg/mL) were applied for interpretation of BMD results [9]. The lowest concentration with complete growth inhibition was considered as the MIC; pinpoint growth was disregarded, and the highest MIC was taken into consideration in case of skipped wells.

The E-test results were read after 20-24 hours of incubation of inoculated plates at 35 ± 2 °C in ambient air. Quality control for AST was performed as per CLSI recommendation using *A. baumannii* ATCC 19606 and *Escherichia coli *ATCC 25922 as control strains [10]. The VITEK 2 Compact (Biomérieux, software version 9.01) test was performed as per the manufacturer's instructions. Both the E-test and VITEK 2 Compact results were interpreted as per the EUCAST 2020 MIC breakpoints for tigecycline against *A. baumannii*. MIC results obtained with the E-test and VITEK 2 Compact were compared with those of BMD to assess inter-method agreement using error-rate-bounded analysis.

Based on BMD results, MIC₅₀ and MIC₉₀ were defined as the MIC values inhibiting 50% and 90% of isolates, respectively. Results obtained by the evaluated methods were compared using modal MICs and geometric mean MICs. Essential agreement (EA) was defined as MIC values obtained by E-test or VITEK 2 Compact falling within ±1 twofold dilution of the BMD MIC. Categorical agreement (CA) was defined as the percentage of isolates showing identical susceptibility categorization compared with BMD. False-resistant and false-susceptible results were classified as major errors (ME) and very major errors (VME), respectively, while discrepancies involving the intermediate category were classified as minor errors (mE).

According to FDA performance criteria, acceptable test performance requires EA and CA ≥ 90% and ME < 2% [11]. Reliability of individual breakpoints was categorized as high when both EA and CA were ≥ 90%, moderate when either EA or CA was ≥ 90%, and low when both EA and CA were <90%. Discrete variables were compared using an unpaired t-test where appropriate, and a p-value <0.05 was considered statistically significant [12].

Ethical considerations

Approval for the study was obtained from the Institutional Ethics Committee of All India Institute of Medical Sciences (AIIMS), Bhubaneswar, prior to commencement (Ref. No. IEC/AIIMS BBSR/PG Thesis/2019-20/08 Dt-15.07.2019).

## Results

During the 24-month surveillance period (May 2019 to June 2021), a total of 28,002 clinical specimens were processed for routine microbiological testing. Among these, 163 (0.58%, 163/28002) were presumptively identified as *Acinetobacter* speciesbased on standard morphological and biochemical characteristics. VITEK 2 Compact confirmation identified 150 of these isolates (92.0%, 150/163) as *Acinetobacter baumannii*. Identification was further confirmed by the detection of the intrinsic blaOXA-51 gene using conventional PCR. These 150 isolates constituted the final study cohort. The remaining 13 isolates (7.97%, 13/163), identified as non-baumannii *Acinetobacter *species (*A. nosocomialis, A. lwoffii,* and *A. pittii*), were excluded from the tigecycline susceptibility analysis, considering their doubtful clinical significance.

The distribution of the 150 *A. baumannii* isolates reflected the typical epidemiology of hospital-associated infections. Respiratory tract specimens accounted for the majority of isolates (54.0%, 81/150), which is consistent with the organism’s association with ventilator-associated infections in intensive care units. Blood culture isolates constituted 17.0% (25/150), indicating invasive bacteraemia. Additional sources included urine (8.0%, 12/150), pus and wound exudates (5.33%, 8/150), and other sterile body fluids (16.0%, 24/150).

BMD was used as the reference method for comparative analysis and interpreted according to EUCAST 2020 *Enterobacterales* breakpoints (susceptible MIC ≤0.5 µg/mL; resistant >0.5 µg/mL), owing to the absence of *A. baumannii*-specific breakpoints [9].

**Figure 1:**
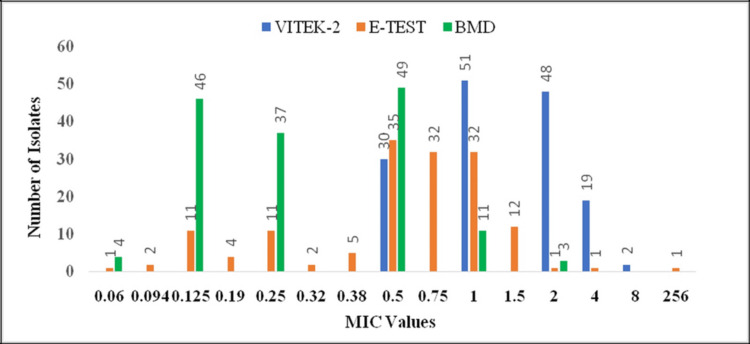
Distribution of minimum inhibitory concentration (MIC) by three different methods: E-test, VITEK 2, and broth microdilution (BMD)

By BMD, 14 of 150 isolates (9.33%) were resistant to tigecycline, while 136 isolates (90.67%) were categorized as susceptible. The MIC distributions of all three methods are depicted in Figure 1. The MIC₅₀ and MIC₉₀ were 0.125 µg/mL and 0.5 µg/mL, respectively, indicating the overall good in vitro activity of tigecycline against the study isolates. MIC distributions obtained by the three methods are shown in Table 1.

**Table 1:** MIC50 and MIC90 of broth microdilution, E-test, and VITEK 2 Compact MIC: Minimum inhibitory concentration; BMD: broth microdilution

Test	MIC_50_	MIC_90_
BMD	0.125 μg/mL	0.5 μg/mL
E-test	0.75 μg/mL	1 μg/mL
VITEK 2 Compact	1 μg/mL	4 μg/mL

The E-test method (HiMedia gradient strips; HiMedia Laboratories Private Limited, Thane, India; MIC range 0.016-256 µg/mL) consistently produced higher MIC values than BMD. Using EUCAST interpretive criteria, 79 of 150 isolates (52.66%) were categorized as resistant by E-test, approximately 5.6-fold higher than the resistance rate obtained by BMD. The MIC₅₀ and MIC₉₀ values determined by E-test were 0.75 µg/mL and 1 µg/mL, respectively, both higher than the corresponding BMD values (Table 1).

**Figure 2:**
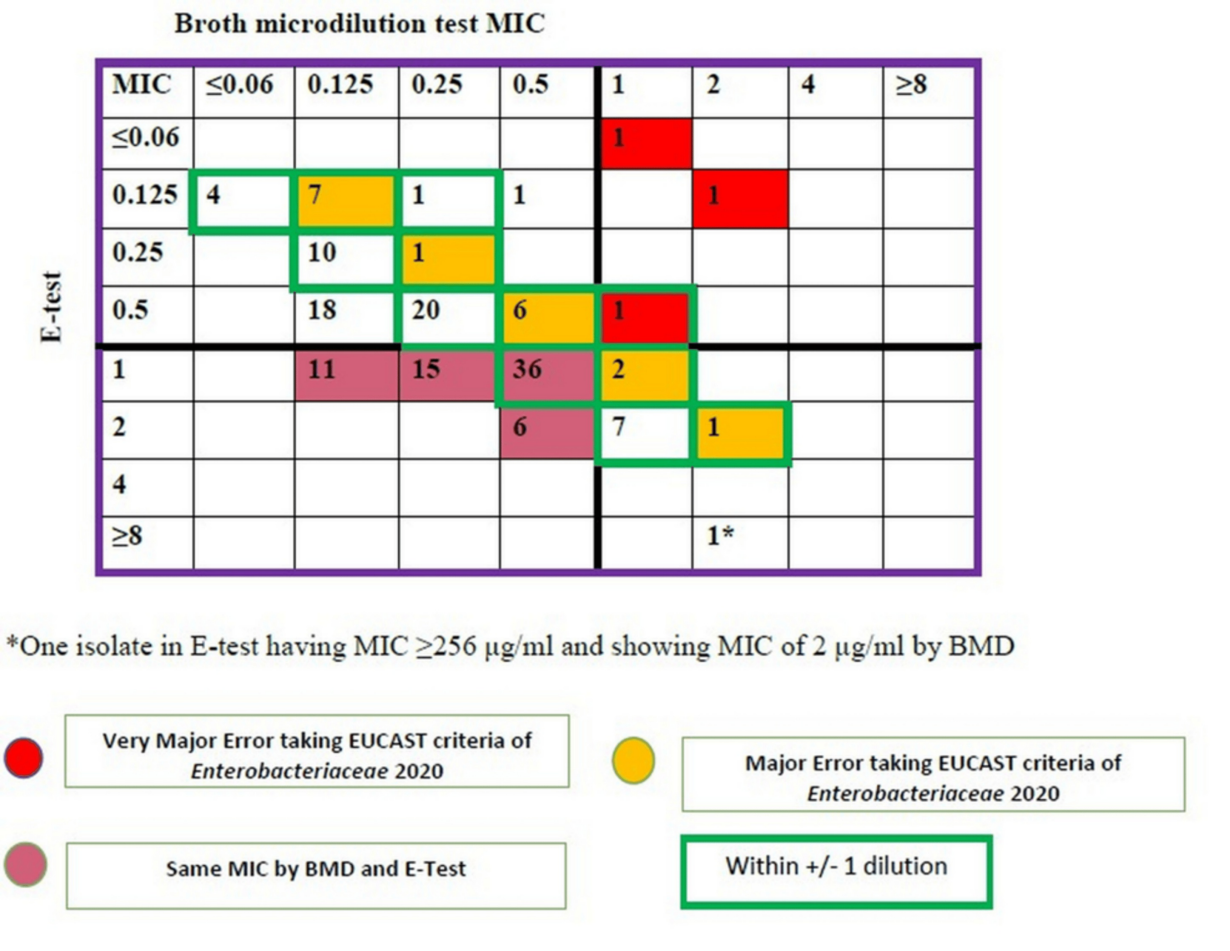
Categorical agreement analysis of minimum inhibitory concentration (MIC) values of tigecycline in broth microdilution vs. E-test EUCAST: European Committee on Antimicrobial Susceptibility Testing; BMD: broth microdilution

Agreement analysis demonstrated limited concordance between the E-test and the BMD (Figure 2). Identical MIC values were observed for 17 isolates (11.33%). Among the remaining isolates, 79 (59.39%) were within ±1 two-fold dilution of the BMD MIC (essential agreement). CA was observed in 79 of 150 isolates (52.66%), substantially below the FDA-recommended threshold of ≥ 90%.

Error analysis revealed unacceptable discrepancies. ME (false resistance) occurred in 68 of 136 BMD-susceptible isolates (50.0%), and VME (false susceptibility) occurred in 3 of 14 BMD-resistant isolates (21.42%). Minor errors were not observed because EUCAST guidelines do not include an intermediate category for tigecycline.

The VITEK 2 Compact showed greater divergence from BMD results. Resistance was detected in 120 of 150 isolates (80.0%), approximately 8.6-fold higher than the BMD resistance rate. The MIC₅₀ and MIC₉₀ values obtained with the VITEK 2 Compact were 1 µg/mL and 4 µg/mL, respectively, both markedly higher than the BMD values, indicating a systematic upward shift in MIC estimation.

**Figure 3:**
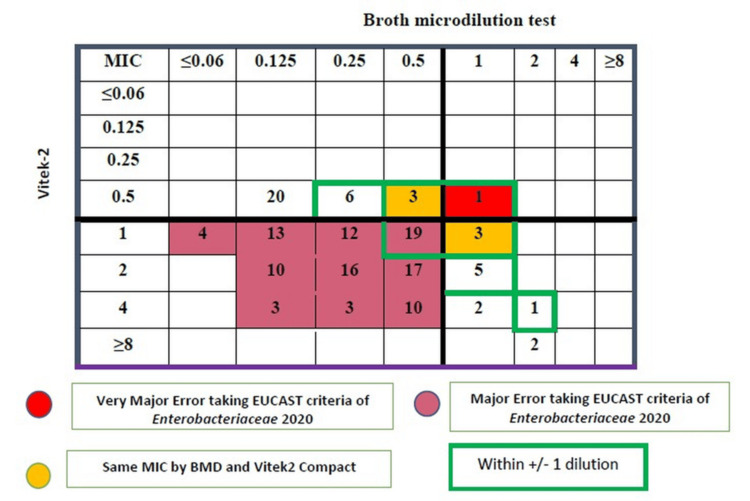
Categorical agreement analysis of minimum inhibitory concentration (MIC) values of tigecycline in broth microdilution vs. VITEK 2 EUCAST: European Committee on Antimicrobial Susceptibility Testing; BMD: broth microdilution

Agreement analysis showed identical MICs between VITEK 2 Compact and BMD in only six isolates. Essential agreement was observed in 38 isolates (25.33%), and CA was achieved in 42 isolates (28.0%) (Figure 3). The ME rate was 78.67% (107/136 BMD-susceptible isolates incorrectly categorized as resistant), while VME occurred in 1 of 14 BMD-resistant isolates (7.14%).

A clear method-dependent bias was observed, with progressive increases in MIC₅₀ and MIC₉₀ values from BMD to E-test to VITEK 2 Compact. Compared with BMD, E-test demonstrated approximately sixfold higher MIC₅₀ values and twofold higher MIC₉₀ values, while VITEK 2 Compact showed approximately eightfold higher values for both parameters.

When evaluated against FDA validation criteria (EA ≥ 90%, CA ≥ 90%, ME < 2%, VME < 2%), both alternative methods demonstrated unacceptable performance. The E-test showed EA 64%, CA 52.66%, ME 50%, and VME 21.42%, whereas VITEK 2 Compact demonstrated EA 25.33%, CA 28%, ME 78.67%, and VME 7.14%, confirming substantial discordance compared with the reference BMD method.

## Discussion

*A. baumannii* has become the foremost cause of nosocomial infections all over the world. Although tigecycline is regarded as a last-resort antibiotic for MDRAB infections, resistance to tigecycline has been reported in India and abroad in recent years [6,8]. So far, very few studies have used the BMD method to conduct susceptibility testing for tigecycline and have reported resistance ranging from 0% to as high as 85% [8,13,14]. Tigecycline susceptibility varies according to the method and interpretative criteria used to read the susceptibility results. As there was no clear-cut clinical breakpoint defined by CLSI, EUCAST, or FDA for *Acinetobacter* spp., the majority of the studies have used either FDA or EUCAST. The breakpoint defined by the FDA is ≤2-Susceptible, 2-8-Intermediate, and ≥8-Resistant [15]. Until 2018, the breakpoints mentioned by EUCAST were ≤1-Susceptible and >2-Resistant. Hence, there were twofold differences in the MICs between EUCAST and FDA for defining resistance [15,16]. However, EUCAST revised the breakpoints in 2019, with MICs ≤0.5 µg/mL considered susceptible and >0.5 µg/mL resistant [17]. Therefore, the breakpoint for tigecycline has been lowered in EUCAST since 2019, resulting in a twofold difference from the FDA cutoff level of≥ 8 µg/mL. Thus, the isolates with MICs of 1-8 µg/mL fall within a gray zone because they are considered susceptible/intermediate by the FDA but resistant according to the EUCAST criteria [6]. EUCAST suggests a dosage of 100 mg followed by 50 mg every 12 hours when the MIC of the isolate is ≤0.5 µg/mL in noncritical patients and for critically ill patients, a high loading dose of 200 mg followed by 100 mg every 12 hours. Pharmacokinetics (PK)/pharmacodynamics (PD) of tigecycline at these high doses predicts that MDR strains with tigecycline MICs of up to 1 µg/mL will respond to treatment [18]. Therefore, in our study, we followed the current EUCAST 2020 guidelines for *Enterobacterales*, with MIC values >0.5 µg/mL considered resistant and ≤0.5 µg/mL susceptible.

Our study revealed tigecycline resistance to be 9.33% (14/150), which was similar to a recent study (9%) in China by Yin et al. and 8% from an Indian study by Kumari et al., but they had followed a different method and used a different breakpoint as well [19,20].

Using the E-test, tigecycline resistance in our study was 52.66%, which is comparable to that reported by Yin et al. (41.6%) from China and by Behera et al. (57.6%) and Shankar et al. (59.8%) from India [6,19,21]. However, very high resistance (74.2%) has been reported from a study from Poland [22]. One plausible explanation for the high MICs by E-test could be a result of heteroresistance of *A. baumannii* isolates to tigecycline [23]. However, further studies are needed to investigate the reasons for high resistance rates on the E-test.

VITEK 2 Compact was also not proven reliable for tigecycline susceptibility, and higher resistance rates have been observed earlier [24]. The present study showed resistance by VITEK 2 Compact at 80%, which was relatively high compared to the other two methods. Few authors from India have reported tigecycline resistance rates using the VITEK 2 Compact at 24.48%-38% [25,26]. However, our study is comparable to that of Vellore et al., who have reported a very high percentage (82.1%) of tigecycline resistance among *A. baumannii* isolates [6]. In our study, the E test showed essential agreement and CA of 64% and 52.66%, respectively, compared with BMD, consistent with a Chinese study reporting CA of 59.3% [19]. However, CA on the E-test as per FDA criteria was found to be 83.9% in both India and Greece [6,24].

The ME of the tigecycline E-test was 50%, and there were no minor errors in our study. The cause for observing no minor error here may be due to the absence of intermediate criteria in the EUCAST breakpoint. As reported by Shankar et al. (FDA criteria), there was no ME; however, a minor error was 16.1% (not acceptable as >10%) [6]. The discordant results between the E-test and BMD were due to the E-test overcalling resistance and hence not correlating with BMD results. The performance of VITEK 2 Compact was even worse than the E-test when compared to BMD. In our study, 25.33% EA and 28% CA were observed, similar to that of Shankar et al. (CA-25% and EA-55.4%) [6]. Although E-test and VITEK 2 Compact are regarded as the two most convenient methods for MIC determination in diagnostic microbiology laboratories compared with BMD, their usefulness for tigecycline susceptibility testing is limited. The results of all these studies indicate that neither the E-test nor the VITEK 2 meets the CLSI recommendation as an alternative to the reference BMD method. According to FDA guidelines, a testing method is considered unreliable when the minor error is >10%, and the ME is >3%, regardless of EA and CA values. Determining the MIC_50_ and MIC_90_ helps identify the epidemiological cutoff values, as the clinical breakpoints for tigecycline against *A. baumannii* are not defined in any available guidelines.

The MIC_50_ and MIC_90_ of tigecycline in our study were 0.125 μg/mL and 0.5 μg/mL, respectively, as determined by the BMD method. The study from Vellore has documented higher tigecycline MIC_50_ (1 µg/mL) and MIC_90_ (2 µg/mL) as compared to ours [27]. Lavrinenko et al. have reported lower MIC_50_ (0.125 µg/mL) and MIC_90_ (0.125 µg/mL) in a study from Kazakhstan [28]. However, the MIC_50_ and MIC_90_ values may vary and depend on the number of isolates tested. We observed higher MIC_50_ and MIC_90_ values on the E-test (0.75 μg/mL and 1 μg/mL, respectively) than on BMD. Several earlier studies using the VITEK 2 Compact also reported higher MIC_50_ and MIC_90 _values (4 μg/mL, 8 μg/mL) [25] and (4 μg/mL, 16 μg/mL) [29].

Discordant MICs by E-test and VITEK 2 Compact with those of the reference BMD are a cause for concern, because many clinical microbiology laboratories use either of these two tests for the tigecycline susceptibility test of *A. baumannii*. The discrepancies in BMD results may be attributed to several factors that influence the performance of the tigecycline susceptibility test, including (i) date of preparation of the medium, (ii) ion content of the medium, and/or (iii) organism expressing heteroresistance to tigecycline. It is hypothesized that the composition of the medium has an effect on tigecycline MICs. The use of freshly prepared (<12 hours) broth is required for the tigecycline BMD test. In particular, high manganese concentration in Mueller-Hinton agar plates has been reported to influence the tigecycline susceptibility with higher MIC values [30]. Very few studies are reported on tigecycline susceptibility testing by the reference BMD method.

## Conclusions

In our setting, the reference BMD method and the routinely used E-test and VITEK 2 Compact yielded discordant results for tigecycline susceptibility testing. Our findings suggest that gradient diffusion (E-test) and automated VITEK 2 Compact systems may significantly overestimate resistance compared to the reference BMD method. This overestimation could potentially lead to unnecessary restriction of an important last-resort antimicrobial agent. Therefore, laboratories performing routine tigecycline susceptibility testing and relying solely on E-test or VITEK 2 Compact results should interpret findings with caution, with BMD considered as a confirmatory method for validation.

The limitations of this study include its single-center design, small sample size, absence of molecular characterization of resistance determinants (such as tet(X) and adeABC), and lack of correlation between MIC categories and clinical outcomes. Hence, larger multicenter studies addressing these limitations are warranted to establish appropriate interpretative categories for Indian *A. baumannii *isolates and to support future updates of susceptibility testing guidelines.
